# Clomipramine causes osteoporosis by promoting osteoclastogenesis via E3 ligase Itch, which is prevented by Zoledronic acid

**DOI:** 10.1038/srep41358

**Published:** 2017-02-01

**Authors:** Xing Li, Wen Sun, Jinbo Li, Mengmeng Wang, Hengwei Zhang, Lingpeng Pei, Brendan F. Boyce, Zhiyu Wang, Lianping Xing

**Affiliations:** 1 Department of Immuno-oncology, Fourth Hospital of Hebei Medical University, Shijiazhuang 050011, China; 2 Department of Pathology and Laboratory Medicine, University of Rochester Medical Center, Rochester, NY 14642, USA; 3 Institute of Chinese Minority Traditional Medicine, MINZU University of China, Beijing 100081, China; 4 Center for Musculoskeletal Research, University of Rochester Medical Center, Rochester, NY 14642, USA

## Abstract

Patients taking antidepressants, including Clomipramine (CLP), have an increased risk of osteoporotic fracture. However, the effects of CLP on bone metabolism are unknown. Here, we demonstrate that WT mice treated with CLP for 2 weeks had significantly reduced trabecular bone volume and cortical bone thickness, associated with increased osteoclast (OC) numbers, but had no change in osteoblast numbers or bone formation rate. Bone marrow cells from CLP-treated mice had normal OC precursor frequency, but formed significantly more OCs when they were cultured with RANKL and M-CSF. CLP promoted OC formation and bone resorption and expression of OC-associated genes. CLP-induced bone loss was prevented by Zoledronic acid. At the molecular level, CLP inhibited the activity of the ubiquitin E3 ligase Itch. CLP did not promote OC formation from bone marrow cells of *Itch−*/*−* mice *in vitro* nor induce bone loss in *Itch−*/*−* mice. Our findings indicate that CLP causes bone loss by enhancing Itch-mediated osteoclastogenesis, which was prevented by Zoledronic acid. Thus, anti-resorptive therapy could be used to prevent bone loss in patients taking antidepressants, such as CLP.

A high risk of fracture was reported in the late 1990s in patients with depression, associated with low bone mineral density (BMD)^1^,^2^. Multiple factors, including poor nutrition, weight loss, muscle weakness, and anti-depression medications^3^,^4^,^5^, could affect bone health of depressed patients. Tricyclic antidepressants (TCAs) and selective serotonin reuptake inhibitors (SSRIs) are two major classes of anti-depression drugs. Vestergaard *et al*. reported that TCAs are associated with a dose-dependent increase in fracture risk in a study of 498,617 cases, but they provided no evidence indicating that TCAs use can affect BMD^6^,^7^. A recent 5 years of follow-up study reported that TCA users had greater annual BMD loss than non-users^8^. Some investigators have speculated that TCA-associated bone fractures are adverse effects of TCAs, such as changes in mental behavior, cardiac dysrhythmias and falls^9^,^10^,^11^. Whether TCAs have a direct effect on bone cell functions remains to be studied.

Clomipramine (Brand name: Anafranil) was the first drug to be approved by the FDA to treat patients with obsessive-compulsive disorders. Since the 1960s it has been also used to treat patients with depression, panic disorders, and chronic pain. Clomipramine (CLP) is an antagonist/inverse agonist for the histamine H1 receptor, the muscarinic acetylcholine receptors and the α1 adrenergic receptor^12^,^13^. CLP is also a tricyclic compound that selectively inhibits the serotonin reuptake. Recent studies indicate that bone cells, including osteocytes, osteoblasts, periosteocytes, and osteoclasts (OCs), express serotonin receptors or transporters^14^,^15^, suggesting that bone is a target tissue of serotonin^16^. However, the effects of serotonin-mediated events in bone cell regulation are controversial^17^ because the data are both positive and negative depending on the experimental settings^18^,^19^,^20^. Patients taking CLP have a high incidence of osteoporotic fracture^6^. Bone mass is maintained by a balance of OC-mediated resorption and osteoblast (OB)-mediated bone formation. However, it is not known if CLP affects OCs or OBs to cause bone loss. This is an important question because if CLP induces bone loss by promoting OC function, one can treat patients who take CLP with anti-resportive drugs.

Bisphosphonates are clinically used anti-resorptive drugs for patients with osteoporosis mainly by promoting apoptosis of mature OCs and inhibiting RANK expression of OC precursors^21^. Zoledronic acid (ZA), a bisphosphonate, has been considered as the most effective drug on treating bone tumor metastasis^22^,^23^ and osteoporosis^24^. We used ZA to prevent bone loss in a mouse model of leukemia^25^.

A recent drug screening study reported that CLP inhibits the activity of ubiquitin E3 ligase Itch^26^. Itch negatively regulates multiple inflammatory signaling pathways, including JNK^27^ and NF-κB^28^ in variety of cell types. We reported previously that OC precursors from *Itch−*/*−* mice have prolonged RANKL-induced NF-κB activation and they formed more mature OCs *in vitro* and *in vivo*^29^, indicating that Itch is a negative regulator of osteoclastogenesis. Finding of CLP inhibiting Itch raises an interesting possibility that CLP may involve in osteoclastogenesis via the regulation of Itch. In this study, we examined the requirement of Itch for CLP-induced bone loss in *Itch−*/*−* mice, and if this is via OCs or OBs and can be prevented by ZA treatment.

## Results

### Mice-treated with Clomipramine have decreased bone volume

**Figure 1:**
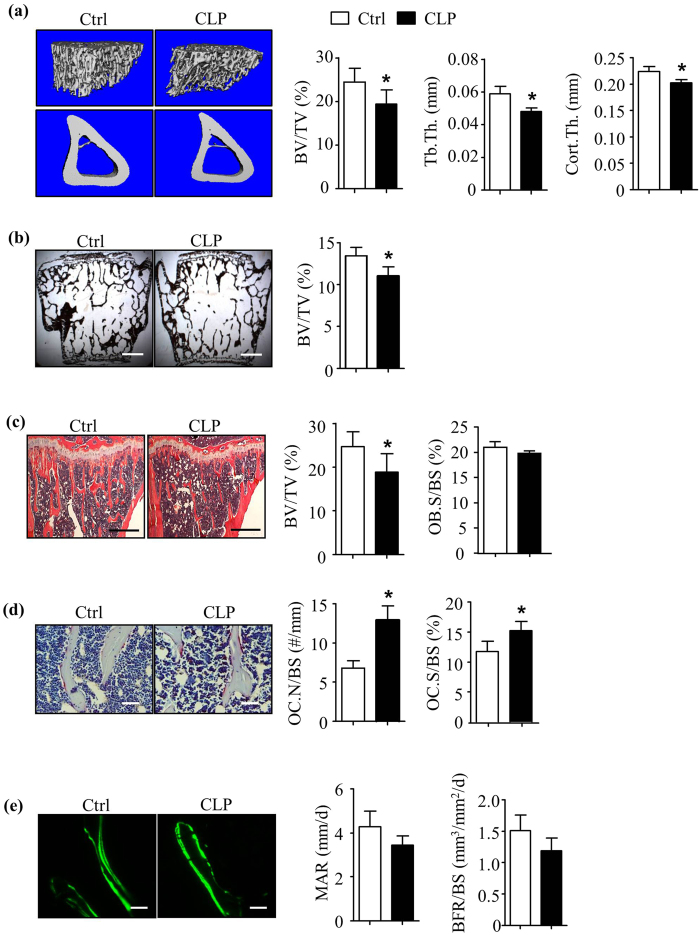
Mice-treated with Clomipramine have decreased bone volume and increased osteoclast numbers. Two-month-old WT C57BL/6 J male mice were treated with Clomipramine (10 mg/kg/intraperitoneal injection) or saline Ctrl daily for 14 days. Mice were sacrificed 4 hours after the last injection. (**a**) Tibiae were subjected to μCT. Representative scans and statistical analysis for tibial trabecular bone volume and cortical bone thickness. (**b**) Von Kossa staining of vertebra. (**c**) H&E-stained tibial sections and histomorphometric analysis of BV/TV (%) and OB.S/BS (%). (**d**) TRAP-stained tibial sections and histomorphometric analysis of OC.S/BS (%) and OC.N/BS (#/mm). (**e**) Dual calcein labeling of tibial trabecular bones and histomorphometric analysis of MAR and BFR. Values are mean ± S.D of 5 to 7 mice/group. *p < 0.05 versus Ctrl. Ctrl: saline, CLP: Clomipramine. Scale bars: 500 μm in (**b**) and (**c**); 50 μm in (**d**) and (**e**).

Clomipramine (CLP) is prescribed to patients with depression or other mental disorders in a wide range of doses (typically 25–250 mg/day. e.g. 12.5–125 mg/m^2^ for a person with 2 m^2^ of body surface), starting at the lowest dose and gradually increasing until an effective dose is reached^30^. To determine the effects of CLP on bone mass, we treated 2-month-old WT C57BL/6 J male mice with CLP at 10 mg/kg daily or with saline Control (Ctrl) for 2 weeks. This dose was selected because it is approximately equivalent to the 31 mg/m^2^ dose administered to patients (for a patient with 2 m^2^ of body surface, the dose is 62 mg/day, which is a lower effective dose used in the clinic)^31^. CLP reduced bone volume in both long bones and vertebrae. Tibial μCTs showed that CLP decreased trabecular bone volume (BV/TV: 19.4% ± 3.3% vs. 24.5% ± 3.1% in Ctrl mice), trabecular thickness (0.048 mm ± 0.002 mm vs. 0.060 mm ± 0.004 mm in Ctrl mice) and cortical bone thickness (0.202 mm ± 0.006 mm vs. 0.224 mm ± 0.010 mm in Ctrl mice) (Fig. 1a). Decreased bone volume (BV/TV: 11.02% ± 1.12% vs. 13.46% ± 0.99% in Ctrl mice) was confirmed in Von Kossa-stained sections of vertebrae (Fig. 1b). CLP did not affect body weight or induce any pathologic changes in sections of internal organs, including liver, spleen and intestine (data not shown).

### Mice treated with Clomipramine have increased osteoclast formation *in vivo*

Under normal conditions, bone remodeling is balanced by osteoclast (OC)-mediated bone resorption and osteoblast (OB)-mediated bone formation. Any reagents and risk factors affecting this balance could cause bone loss or excessive bone formation. To investigate if bone loss induced by CLP is mediated by effects on OCs, OBs or both, we performed histomorphometric analysis of bone sections from CLP- and vehicle-treated mice. Analysis of H&E-stained tibial sections showed significantly decreased bone volume (BV/TV: 18.8% ± 3.3% vs. 24.7% ± 2.4% in Ctrl mice), but no change in OB parameters (OB surface/bone surface: 19.9% ± 0.4% vs. 21.3% ± 1.2% in Ctrl mice; Fig. 1c). TRAP-Fast green-stained sections showed significantly increased OC parameters (OC surface/bone surface: 15.3% ± 1.5% vs. 11.8% ± 1.5% in Ctrl mice; No. OCs/mm bone surface: 12.9 ± 2.7 vs. 6.8 ± 0.9 in Ctrl mice) (Fig. 1d). The double calcein labeling showed no significant differences in BFR/BS in bone sections from mice treated with CLP compared to Ctrl mice (1.51 ± 0.25 vs. 1.18 ± 0.21 μm^3^/μm^2^/d, *p* = 0.3359) (Fig. 1e).

### Bone marrow cells from mice-treated with Clomipramine have increased osteoclast forming potential *ex vivo*

**Figure 2:**
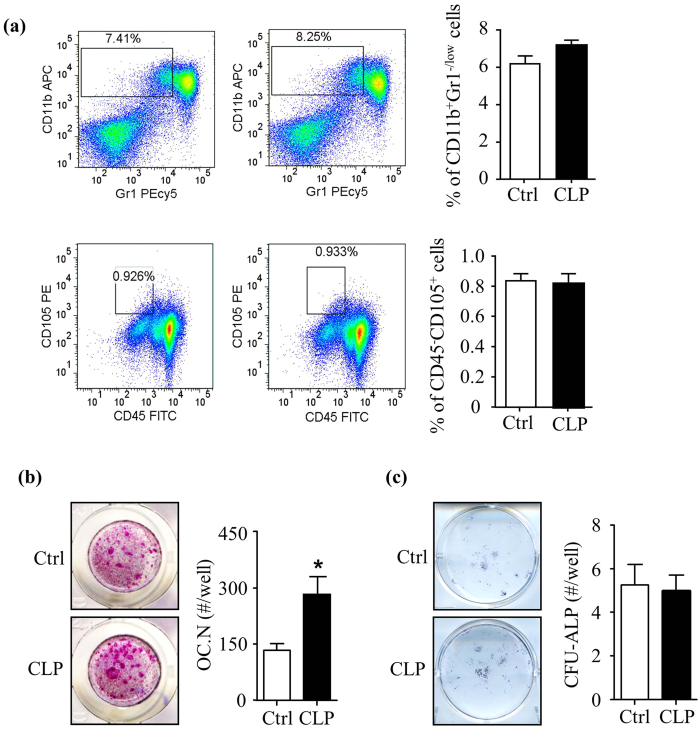
Bone marrow cells from mice treated with Clomipramine have increased osteoclast forming potential *ex vivo*. WT mice were treated with Clomipramine, as in Fig. 1. Bone marrow cells were used. (**a**) Cells were stained with anti-CD11b and Gr1- Abs for the percentage of CD11b^+^Gr1^−/low^ OC precursors or anti-CD45 and CD105 Abs for the percentage of CD45^-^CD105^+^ mesenchymal progenitors. Values are mean ± S.D. of 5 to 7 mice/group. (**b**) Cells were cultured with M-CSF and RANKL for 7 d in OC formation assays and stained for TRAP activity. Photos show TRAP^+^ OCs. TRAP^+^ OC number was assessed. Values are mean ± S.D. of 4 wells/treatment. Experiments = 3. (**c**) Cells were cultured in osteoblast-inducing medium for 21 d. The number of CFU-ALP^+^ colonies was counted. Values are mean ± S.D. of 4 wells/treatment. Experiments = 3. *p < 0.05 versus Ctrl. Ctrl: saline, CLP: Clomipramine.

To determine if CLP affects OC precursor frequency in bone marrow (BM), we stained BM cells from CLP-treated mice with cell surface markers for OC precursors and mesenchymal progenitors and subjected them to flow cytometric analysis. We defined OC precursors as CD11b^+^Gr1^−/low^ cells and mesenchymal progenitors as CD45^−^CD105^+^ cells^32^,^33^. We did not detect any difference in the percentage of CD11b^+^Gr1^−/low^ and CD45^−^CD105^+^ cell populations between CLP-treated and Ctrl mice (CD11b^+^Gr1^−/low^ cells: 7.1% ± 0.2% vs. 6.1% ± 0.3% in Ctrl mice; CD45^−^CD105^+^ cells: 0.81% ± 0.03% vs. 0.83% ± 0.02% in Ctrl mice) (Fig. 2a). When we cultured these BM cells in our OC formation assays, we found that cells from CLP-treated mice formed many more OCs and OC area than Ctrl mice (0.81 mm^2^ ± 0.12 mm^2^ vs. 0.31 mm^2^ ± 0.03 mm^2^) (Fig. 2b). In contrast, BM cells from CLP-treated mice had no significant changes in OB differentiation as indicated by CFU-ALP colony formation assays (5.1 ± 0.7 vs. 5.3 ± 1.0 in Ctrl mice) (Fig. 2c).

### Clomipramine increases osteoclast formation and bone resorption *in vitro*

**Figure 3:**
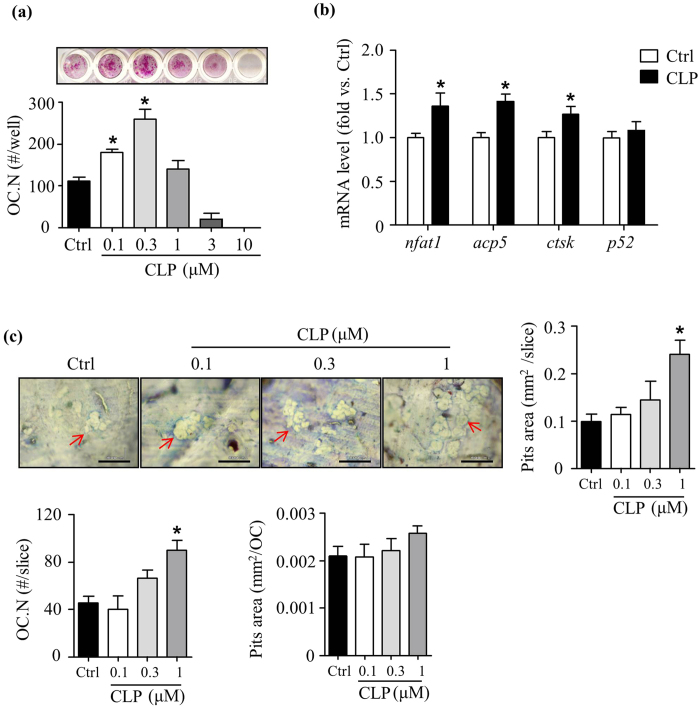
Clomipramine increases osteoclast formation and bone resorption *in vitro* . Bone marrow cells from WT mice were used. (**a**) Cells were treated with indicated concentrations of CLP in the presence of RANKL and M-CSF for 7 days in OC formation assays. Cells were stained for TRAP activity. TRAP^+^ OC number was assessed. Values are mean ± S.D. of 4 wells/treatment. Experiments = 3. (**b**) Cells were cultured with RANKL and M-CSF and treated with CLP (0.3 μM) for 7 days. The expression levels of OC-related genes were determined by qPCR. Fold changes in relative gene expression were calculated in relation to expression levels in Ctrl. Experiments = 3. (**c**) Bone resorption assays were performed by culturing cells on bone slides for 10 days using the same culture conditions, as in (**a**). TRAP staining was performed for counting OC number. OCs on bone slices were then removed and followed by toluidine blue staining for measuring resorption pits. Bar graphs showing pits area and OC number per bone slice, and pits area per OC, respectively. Values are mean ± S.D. of 4 bone slices. Experiments = 3. *p < 0.05 versus Ctrl. Ctrl: PBS, CLP: Clomipramine. Scale bars: 100 μm in (**c**).

To determine if CLP directly affects OC formation and function, we cultured BM cells from WT mice with M-CSF and RANKL for 7 days in the presence of different concentrations of CLP (0–10 μM). We found that CLP significantly increased OC numbers (Fig. 3a) and area, at a dose of 0.1 μM and 0.3 μM (13.3 mm^2^ ± 0.8 mm^2^ vs. 6.06 mm^2^ ± 1.3 mm^2^ in Ctrl). The expression levels of OC-related genes, including *nfatc1, acp5* and *ctsk*, were significantly increased in RNA extracted from CLP-treated cells (Fig. 3b). In addition, CLP increased total OC numbers and resorption area per bone slice when they were cultured on the bone slices, but not pit area/OC (Fig. 3c).

### Zoledronic acid treatment prevents Clomipramine-induced bone loss

**Figure 4:**
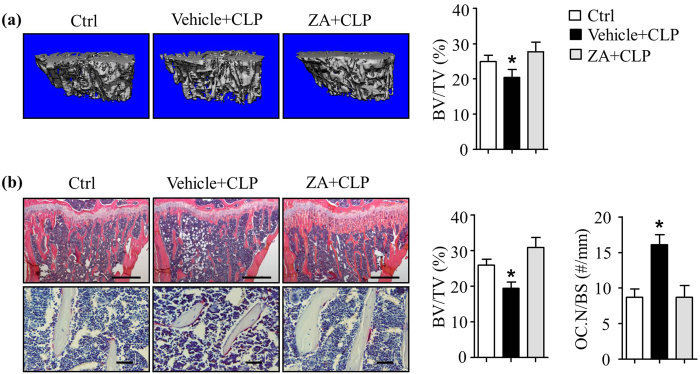
Zoledronic acid prevents Clomipramine-induced bone loss . WT mice were treated with Zoledronic acid (n = 6) or saline (n = 5) or for 14 days followed by Clomipramine for an additional 14 days. In a separate group, mice were treated with water (vehicle for Clomipramine) for 14 days (n = 4) followed by saline (vehicle for Zoledronic acid) for an additional 14 days. Mice were sacrificed 4 hours after last injection. (**a**) Tibiae were subjected to μCT. (**b**) H&E and TRAP-stained tibial sections and histomorphometric analysis of BV/TV (%) and OC.N/BS (#/mm). Values are mean ± S.D. of 4–6 mice/group. *p < 0.05 versus Ctrl and CLP + ZA-treated groups. Scale bars: 500 μm in upper and 50 μm in lower panel in (**b**). Vehicle + CLP: saline + CLP, ZA + CLP: Zoledronic acid + Clomipramine, Ctrl: water + saline.

Bisphosphonates are used to inhibit bone resorption in patients with osteoporosis^34^. Because our data suggest that bone loss caused by CLP treatment is due mainly to increased OC-mediated bone resorption, we next tested if zoledronic acid (ZA), a commonly used bisphosphonate, could prevent the reduction in bone mass caused by CLP. We treated WT mice with ZA (0.25 mg/kg, i.p. twice/week) or sterile water for 2 weeks^25^ and followed with CLP treatment for an additional 2 weeks. Compared to mice that were treated with sterile water (vehicle for ZA) followed by saline (vehicle for CLP), CLP treated mice developed bone loss and increased OC numbers as showed in Fig. 1. ZA restored CLP-reduced bone volume (Fig. 4a). Analysis of H&E- and TRAP-stained sections revealed that ZA restored CLP-induced bone loss and OC formation (Fig. 4b).

### Mice deficient in the ubiquitin E3 ligase, Itch, do not develop Clomipramine-induced bone loss

**Figure 5:**
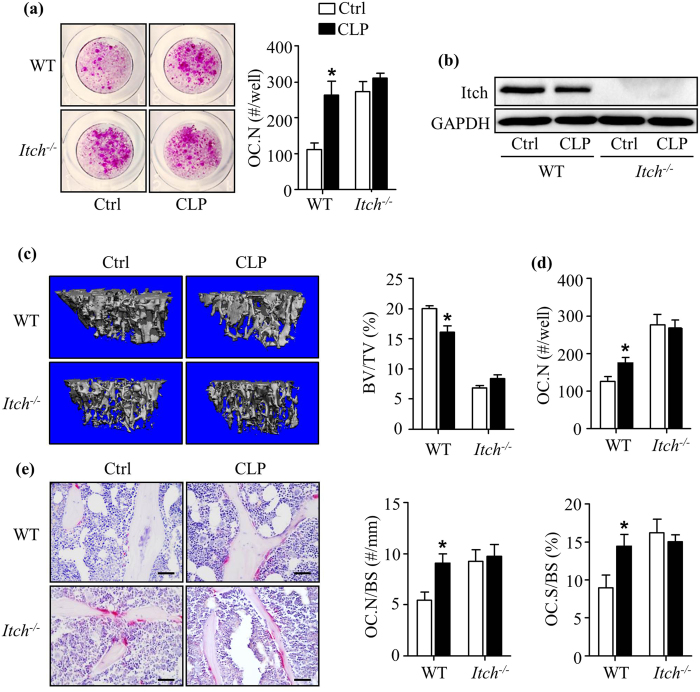
Clomipramine does not induce bone loss in mice deficient in the ubiquitin E3 ligase, Itch . *Itch−*/*−* mice (3-month-old, male) and their WT littermates were treated with CLP (10 mg/kg/intraperitoneal injection) or saline Ctrl daily for 14 days. (**a**) Bone marrow cells were cultured in an OC formation assay, as in Fig. 3, with or without CLP treatment. TRAP^+^ OC number was measured. Values are mean ± S.D. of 4 wells. Experiments = 3. (**b**) Itch protein expression levels in OCs were examined by Western blot analysis. Experiments = 3. (**c**) *Itch−*/*−* mice and WT littermates (n = 4–5/genotype) were treated with CLP or water for 14 days, as in Fig. 1. Tibiae were subjected to μCT. Representative scans and statistical analysis for fold changes of tibial trabecular bone volume. Values are mean ± S.D. of 4–5 mice. (**d**) Bone marrow cells from treated mice were cultured in OC formation assays. TRAP ^+^OC number was assessed. Values are mean ± S.D. of 4 wells. (**e**) TRAP-stained tibial sections and histomorphometric analysis. Values are mean ± S.D. of 4–5 mice. *p < 0.05 versus Ctrl. Scale bars: 50 μm in (**e**). Ctrl: water, CLP: Clomipramine.

The molecular mechanisms whereby CLP induces its antidepressant effects have not been well investigated. Some early reports indicate that CLP may affect serotonin uptake^35^, a Rho-kinase pathway and Ca(2+)-channels^36^. A more recent study using high throughput screening for inhibitors of the ubiquitin E3 ligase, Itch, indicates that CLP can specifically inhibit Itch ligase activity by binding irreversibly to HECT domains or catalytic cysteines^26^. Interestingly, we reported that Itch negatively regulates OC formation^29^. Thus, we hypothesized that *Itch−*/*−* mice would not develop CLP-induced osteoclastogenesis and bone loss. We treated BM cells from WT and *Itch−*/*−* mice with CLP in our OC formation assays and found that *Itch−*/*−* cells formed more OCs than WT cells (7.9 ± 1.35 mm^2^ vs. 3.0 ± 0.65 mm^2^)^29^. However, CLP treatment increased OC formation from WT cells (6.4 ± 0.75mm^2^ vs. 3.0 ± 0.65mm^2^), but not in *Itch−*/*−* cells (Fig. 5a and b). At basal levels, *Itch−*/*−* mice have low bone mass and high OC number compared to WT mice because itch is a negative regulator of OC formation as we previously reported^29^. We found that CLP administration did not cause bone loss in *Itch−*/*−* mice, but it caused bone loss in WT littermates (Fig. 5c). BM cells from CLP-treated WT mice formed more OCs when they were cultured with RANKL and M-CSF than cells from vehicle-treated WT mice, but this effect was not observed in cells from CLP-treated *Itch−*/*−* mice (Fig. 5d). Consistently, CLP increased OC numbers and surface area in WT mice, but not in *Itch−*/*−* mice (Fig. 5e).

## Discussion

Antidepressant medications are primarily prescribed for depression and anxiety^37^,^38^ and for some other non-psychiatric conditions^39^. The effects of antidepressants on bone have not been studied in depth, and the findings of some reports are controversial. For example, the antidepressant, lithium, accelerates proliferation of human bone marrow-derived mesenchymal progenitor cells *in vitro*, associated with inhibition of GSK-3β and accumulation of β-catenin^40^. Local application of lithium promotes bone regeneration by affecting both osteoblasts and osteoclasts (OCs)^41^. In contrast, 5-hydroxytryptophan, the precursor of serotonin, causes bone loss by stimulating osteoclastogenesis via RANKL^42^. In the current study, we investigated the bone’s effects of Clomipramine (CLP), a drug used to treat patients with obsessive-compulsive disorder, major depression, panic attacks, anxiety disorders and premature ejaculation. We found that CLP directly stimulates OC formation and causes bone loss in mice via the ubiquitin E3 ligase, Itch, and that Zoledronic acid (ZA) prevents CLP-induced bone loss. These findings suggest that ZA or other anti-resorption drugs may prevent bone loss and reduce fracture risk in patients who take CLP and other antidepressants.

OC formation requires the RANKL/RANK signaling. RANKL and RANK interactions recruits TNF receptor-associated factor 6 (TRAF6), leading to activation of essential OC transcription factors including NF-κB^43^,^44^. Itch is a negative regulator of NF-κB signaling in lymphocytes and macrophages^28^,^45^,^46^ and it inhibits OC formation by promoting deubiquitination of TRAF6 and suppressing NF-κB signaling^29^. Rossi *et al*. used an ELISA-based high throughput screening assay and screened more than 20,000 compounds (1040 compounds from the NINDS library and ~20,000 compounds from various commercial suppliers) for Itch inhibitors. CLP was the only compound with high affinity that inhibited Itch ligase activity. We found that Itch deficiency abolished CLP-promoted osteoclastogenesis and bone loss, suggesting that Itch inhibition is one of the mechanisms by which CLP induces bone loss.

CLP inhibits the reuptake of serotonin in the central nervous system through interaction with serotonin 5-HT2 and 5-HT3 receptor subtypes^47^,^48^. Thus, it is possible that CLP treatment leads to elevated circulating serotonin levels to inhibit osteoblast function because serotonin produced in the gut negatively regulates osteoblasts^20^. Our study cannot rule out the potential contribution of the serotonin pathway to CLP-induced bone loss. However, we did not detect significant influence of CLP in osteoblast parameters both *in vivo* and *ex vivo*, suggesting that CLP unlikely inhibits osteoblast function. In contrast, CLP increased OC-mediated bone resorption, strongly indicating that CLP affects mainly OCs. CLP promoted OC formation on plastic dishes peaked at 0.3 μM and OC stimulatory effect declined thereafter. CLP stimulated OC formation and bone resorption on bone slices at 1 μM. Our explanation for this difference is that bone slices provide survival factors, making OCs resistant to potential toxic effect of high dose CLP on cells.

CLP is available to patients as capsules for oral administration. We used the daily intraperitoneal injection in our study. This treatment design raises two questions. One is if the serum concentration of the drug in our mice is comparable exposures to humans. The plasma half-life of CLP in mice after intraperitoneal injection is about 2 hours, which is much shorter than in man (about 22 hours) after oral administration^49^. However, even with much shorter half-live, we still observed significantly increased OCs and reduced bone volume in CLP-treated mice, suggesting that it is a potential stimulator of osteoclastogenesis *in vivo*. Another question is the rationale for 2 weeks of CLP dosing. CLP has been used in many mouse behavior studies^13^,^35^. In C57BL/6 J mice, 2 weeks treatment of CLP exerts anti-depression effect^50^, suggesting 2-week dosing may represent a reasonable regimen to study the *in vivo* effect of CLP. Because anti-depressants are often given chronically, we suspect that longer duration of CLP treatment will cause more severe bone loss.

Few studies have addressed the management of fracture risk in patients who take anti-depressants. We found that ZA could reduce bone loss in CLP-treated mice. ZA is used to treat a number of diseases associated with elevated bone resorption, including osteoporosis, osteolytic bone metastases, and multiple myeloma. ZA or other anti-resorption drugs could therefore be useful for prevention of bone loss in antidepressant users. The clinical significance of our study is twofold. It first suggests that monitoring bone mineral density and serum bone resorption markers should be included as components of standard care for patients who take antidepressant medication. It also suggests that anti-resorptive medication may have beneficial effects in these patients.

In summary, we found that CLP, one of the tricyclic antidepressants used for treating patients with depression, anxiety, and obsessive-compulsive disorders, caused decreased bone mass in mice. We found that CLP, an inhibitor of the E3 ligase, Itch, increased OC formation *in vivo* and *in vitro*, and slightly increased osteoblast formation *in vitro*, but not *in vivo*. ZA prevented CLP-induced bone loss in mice. Hence, anti-resorptive drugs may be beneficial for patients on antidepressant drugs who are at risk for osteoporotic fractures.

## Materials and Methods

### Ethics statement

All experiments with animals were performed in strict accordance with the Animal Experimentation Guidelines of the National Institute of Infectious Diseases, and the protocol was approved by the University of Committee on Animal Resources in the University of Rochester.

### Mice and treatment

Two-month-old wild-type C57BL/6 J male mice were purchased from Jax laboratory. *Itch−*/*−* mice on a C57BL/6 J background were generated and genotyped by PCR^51^. Although both male and female patients take anti-depressants, we used male mice in the study because we wanted to avoid the potential influence of estrogen on bone in female mice. Three sets of experiments were performed. 1) To study the role of Clomipramine (CLP, SIGMA, C-7291) on bone mass, mice (n = 5–7/group) were treated with water as control (Ctrl) or CLP (10 mg/kg body weight) by intraperitoneal (i.p.) injection daily for two weeks. 2) To study the effect of Zoledronic acid on CLP-induced bone loss, mice were pre-treated with saline or Zoledronic acid^25^ (ZA, SIGMA, SML0223; 0.25 mg/kg body weight) by i.p. injection twice/week for two weeks, then followed by CLP for another 2 weeks (n = 4–6/group). 3) To study if itch is required for CLP-induced bone loss, age- (3-month-old) and gender- (male) matched *Itch−*/*−* mice (n = 4–5/group) were treated with CLP (10 mg/kg body weight) by i.p. injection daily for two weeks. Mice were sacrificed 4 hours after the last treatment and samples were harvested for analyses.

### μCT, histology, and histomorphometric analysis

Soft tissues were removed from left tibiae and lumbar 1 (L1) vertebrae, fixed in 10% formalin at 4 °C for 24–48 hours on a rocker and transferred to 70% ethanol until μCT scanning. Specimens were scanned at high resolution (10.5 μm) on a VivaCT 40 μCT scanner (Scanco Medical) using 300 ms integration time, 55 kVp energy and 145 μA intensity. Then these tibiae were decalcified in 10% EDTA at 4 °C for 21 days after formalin fixation and embedded in paraffin. Sections (4 μm thick) were stained with H&E for bone volume and osteoblast (OB) analysis and with TRAP-Fast Green for osteoclast (OC) analysis using OsteoMeasure system. OB and OC numbers and surfaces per millimeter bone surface were analyzed in tibial sections with point counting using an eyepiece grid and an Olympus TH4-100 microscope. Tibial and vertebral bone volumes were analyzed in digital slides generated using an Olympus VS120 image analysis system and automated analysis and algorithms that we developed using a Visiopharm Image Analysis System^52^.

### Double calcein labeling

Calcein was administered to mice (10 mg/kg body weight) by i.p. injection at 3 days and 1 day before they were sacrificed. Vertebrae (L4) were harvested, fixed in 10% formalin at 4 °C for 24–48 hours, and embedded in LR white acrylic resin. Bone sections of L4 (4 μm thickness) were cut and observed under fluorescence microscopy. Bone formation rate (BFR) were analyzed using an OsteoMetrics image analysis software system (OsteoMetrics)^32^,^52^.

### Flow cytometry

Bone marrow (BM) cells were flushed from left femora with PBS. Red blood cells were lysed using lysis buffer (Thermo Fisher Scientific, A1049201). After being washed with PBS, cells were suspended in PBS with 2% FBS. Cells were stained with various fluorescein-labeled Abs and subjected to flow cytometric analysis using a Becton-Dickinson FACSCanto II Cytometer. FITC-anti-CD45, PE-anti-CD105, APC-anti-CD11b, and PECY5-anti-Gr1 antibodies were purchased from eBioscience. Results were analyzed by Flowjo7 data analysis software (Ashland, OR).

### Cell culture and analysis

For OC formation assays, BM cells were harvested from right femora and cultured in 96-well plates (5 × 10^4^cells/well) in α-MEM with 10% FBS, 1:50 dilution of conditioned medium containing M-CSF (from an M-CSF producing cell line) and a sub-optimal concentration of RANKL (1 ng/ml) in the presence of different doses of CLP for 7 days. After multinucleated cells were observed under inverted microscopy, cells were fixed by 10% formalin and stained for TRAP activity. TRAP-positive cells with more than 3 nuclei were counted for TRAP^+^ OC number using standard stereology methods^33^. For OC bone resorption assays, BM cells were cultured on sterile cow bone slices (size: 2 mm × 3 mm; thickness: 0.75 mm) under the same conditions for 10 days as described above. Cells were removed and bone slices were stained with 0.1% toluidine blue to visualize resorption pits. The area of pits was quantified and data were expressed as area of pits (mm^2^)/slice. For CFU-Alkaline phosphatase (ALP)-colony formation assays, BM cells from CLP treated mice or from Ctrl mice were cultured in 6-well plates (2 × 10^6^ cells/well) in α-MEM containing 15% FBS with 50 μg/ml ascorbic acid and 10 mM β-glycerophosphate for 21 days. Cells were fixed with 70% ethanol and stained for ALP activity. The number of ALP ^+^colonies with more than 20 cells was counted, as we described previously^32^.

### Quantitative Real Time PCR

Total RNA was extracted from OCs using TRIzol reagent (Invitrogen) according to the manufacturer’s instructions. cDNA was reversely transcribed using the iSCRIPT cDNA Synthesis kit (Bio-Rad) from 1 μg RNA in a 20 μl reaction. qPCR was performed in the iCycler real time PCR machine (Bio-Rad) with iQ SYBR SuperMix (Bio-Rad) according to the manufacturer’s instruction. The expression levels of OC-related genes were examined as we described previously^52^,^53^,^54^. Primer sequences are as follows: nuclear factor of activated T cells c1 (*nfatc1)*, forward, 5′-CACATTCTGGTCCATACGA-3′, and reverse, 5′-CGTGTAGCTGCACAATGG-3′; tartrate-resistant acid phosphatase type 5 (*acp5)*, forward, 5′-TCCTGGCTCAAAAAGCAGTT-3′, and reverse, 5′-ACATAGCCCACACCGTTCTC-3′; cathepsin K (*ctsk),* forward, 5′-CAGCTTCCCCAAGATGTGAT-3′, and reverse, 5′-GAAGCACCAACGAGAGGA-GA-3′; *p52*, forward, 5′-CTGTCAAGATCTGTAACTATGA-3′, and reverse, 5′-ATGTCCTTGGGTCCTACAG-3′; *gapdh*, forward, 5′-GGTCGGTGTGAACGGATTTG-3′, and reverse, 5′-ATGAGCCCTTCCACAATG-3′. Each sample was prepared in triplicate. The relative abundance of each gene was calculated by subtracting the CT value for an individual gene in each sample from the corresponding CT value of *Gapdh* (ΔCT). ΔΔCT values were obtained by subtracting the ΔCT of the reference point. These values were then raised to the power 2 (2ΔΔCT) to yield fold-expression relative to the reference values.

### Western Blot analysis

Whole cell lysates were extracted from cultured OCs using RIPA lysis buffer (Millipore, MA) containing a protease inhibitor cocktail (Roche, Basel, Switzerland) and 20 μg protein per lane was loaded in 10% SDS-PAGE gels, blotted with primary monoclonal antibodies to Itch (1:1000, BD Transduction Laboratories, cat #611198) and GAPDH (1:500, Santa Cruz, cat #32233) overnight, and followed by secondary goat anti-mouse IgG-HRP (1:1000, Bio-Rad, cat #170-6516). Bands were visualized using enhanced chemiluminescence.

### Statistical analysis

All results are listed as mean ± SD. Statistical analysis was performed using GraphPad Prism 5 software (GraphPad Software Inc., San Diego, CA). Comparisons between 2 groups were analyzed using a 2-tailed unpaired Student’s t test. Comparisons among 3 or more groups were carried out using one-way ANOVA followed by Dunnett’s post-hoc multiple comparisons. p values < 0.05 were considered statistically significant.

## Additional Information

**How to cite this article**: Li, X. *et al*. Clomipramine causes osteoporosis by promoting osteoclastogenesis via E3 ligase Itch, which is prevented by Zoledronic acid. *Sci. Rep.* **7**, 41358; doi: 10.1038/srep41358 (2017).

**Publisher's note:** Springer Nature remains neutral with regard to jurisdictional claims in published maps and institutional affiliations.
